# How does the presence of a surgical face mask impair the perceived intensity of facial emotions?

**DOI:** 10.1371/journal.pone.0262344

**Published:** 2022-01-13

**Authors:** Maria Tsantani, Vita Podgajecka, Katie L. H. Gray, Richard Cook

**Affiliations:** 1 Department of Psychological Sciences, Birkbeck, University of London, London, United Kingdom; 2 School of Psychology and Clinical Language Sciences, University of Reading, Reading, United Kingdom; 3 Department of Psychology, University of York, York, United Kingdom; University Hospitals Tubingen: Universitatsklinikum Tubingen, GERMANY

## Abstract

The use of surgical-type face masks has become increasingly common during the COVID-19 pandemic. Recent findings suggest that it is harder to categorise the facial expressions of masked faces, than of unmasked faces. To date, studies of the effects of mask-wearing on emotion recognition have used categorisation paradigms: authors have presented facial expression stimuli and examined participants’ ability to attach the correct label (e.g., happiness, disgust). While the ability to categorise particular expressions is important, this approach overlooks the fact that expression intensity is also informative during social interaction. For example, when predicting an interactant’s future behaviour, it is useful to know whether they are slightly fearful or terrified, contented or very happy, slightly annoyed or angry. Moreover, because categorisation paradigms force observers to pick a single label to describe their percept, any additional dimensionality within observers’ interpretation is lost. In the present study, we adopted a complementary emotion-intensity rating paradigm to study the effects of mask-wearing on expression interpretation. In an online experiment with 120 participants (82 female), we investigated how the presence of face masks affects the perceived emotional profile of prototypical expressions of happiness, sadness, anger, fear, disgust, and surprise. For each of these facial expressions, we measured the perceived intensity of all six emotions. We found that the perceived intensity of intended emotions (i.e., the emotion that the actor intended to convey) was reduced by the presence of a mask for all expressions except for anger. Additionally, when viewing all expressions except surprise, masks increased the perceived intensity of non-intended emotions (i.e., emotions that the actor did not intend to convey). Intensity ratings were unaffected by presentation duration (500ms vs 3000ms), or attitudes towards mask wearing. These findings shed light on the ambiguity that arises when interpreting the facial expressions of masked faces.

## Introduction

In 2020, many governments around the world introduced the mandatory wearing of face masks in public settings in an attempt to mitigate the spread of coronavirus (COVID-19). Within a few months, mask-wearing became the new norm in many countries. In the UK, 98% of adults reported the use of a face covering at the start of May 2021, compared with just 29% at the end of May 2020 [1, 2]. It is possible that the incidence of mask-wearing will remain above pre-pandemic levels for many years to come, as people around the world seek to limit the transmission of new variants. In light of this dramatic change in our day-to-day behaviour, it is important to understand the implications of mask-wearing for non-verbal communication and social interaction.

Faces convey a wealth of information useful for social interaction. Facial cues support inferences about a person’s identity, gender, and age [3, 4]. Similarly, we spontaneously infer the possible character traits of others based on their facial appearance [5, 6]. During social interaction, however, facial expressions are a particularly important form of non-verbal communication that can be used to infer someone’s emotional state and likely intentions [7–9]. In light of their significance, it is no surprise that the recognition of facial expressions benefits from perceptual expertise. Previous research suggests that human adults classify expressions of the so-called ‘basic emotions’ (happiness, sadness, anger, fear, disgust, surprise) with moderate-to-high accuracy (ranging from around 60% to over 90%) after just 200ms of exposure to a face [10, 11].

A number of studies have attempted to pinpoint the critical facial features for the identification of different emotional expressions by occluding parts of the face using the Bubbles technique [12–14], or by presenting faces with shapes covering the eyes or mouth [15–21]. However, with the exception of happiness, the recognition of which is consistently associated with information from the mouth region, the critical features identified for each emotion tend to vary across studies, suggesting that these features are influenced by contextual factors, such as the experimental design and stimuli [22]. The face masks worn during the pandemic typically leave the eye-region visible, but occlude the lower portion of the face [23, 24]. The widespread use of face masks therefore presents an opportunity to study the relative importance of the lower portion of the face in the identification of different emotions using naturalistic stimuli in a socially-relevant context.

There is growing evidence that the presence of face masks hinders the recognition of facial expressions [25]. In a survey of UK adults on the impact of face masks on communication, participants reported difficulties interpreting the facial expressions of mask-wearers [26]. Several studies have also found that observers find it harder to categorise the facial expressions of masked faces, than of unmasked faces [23, 24, 27, 28]. For example, Noyes and colleagues [24] presented facial stimuli that were either angry, disgusted, fearful, happy, sad, surprised, or emotion neutral, for 1 sec. When the expression stimuli were presented unmasked, mean categorisation accuracy was higher (80.5%), than when faces were shown with a face mask (61.5%). The presence of a mask impaired recognition of anger, disgust, fear, happiness, and surprise.

To date, studies of the detrimental effects of mask-wearing on emotion recognition have almost exclusively used categorisation paradigms [23, 24, 27, 28] (although see [29]). Typically, authors have presented facial expression stimuli and examined participants’ ability to attach the correct label (e.g., happiness, disgust, etc). While the ability to categorise particular expressions is important, this approach overlooks the fact that expression intensity is also informative during social interaction. For example, when predicting an interactant’s future behaviour, it is useful to know whether they are slightly fearful or terrified, contented or very happy, slightly annoyed or angry.

Moreover, the categorisation paradigms used previously assume that facial expressions can be sorted into discrete categories (e.g., anger, disgust, fear, happiness, sadness, surprise). In reality, many expressions do not fall neatly into one category or another. Facial expressions of an intended emotion (e.g., anger) can also convey a number of other emotions at the same time (e.g., disgust, fear), which are perceived with varying degrees of intensity [30–32]. The perceived intensity of these non-target emotions can also alter how we respond to an interactant. For example, a surprised expression might elicit a very different response when accompanied by subtle signs of happiness and fear, respectively [33]. Because emotion labelling paradigms force observers to pick a single label to describe what they have seen, this additional dimensionality within observers’ interpretation is lost.

The present study sought to build on previous expression categorisation research by adopting a complementary emotion-intensity rating paradigm to further understand the effects of mask-wearing on expression interpretation. We sought to investigate how the presence of a surgical-type face mask affects the perceived emotional profile of prototypical facial expressions of happiness, sadness, anger, fear, disgust, and surprise, and neutral expressions. For each of these facial expressions, we measured the perceived intensity of all six basic emotions. On the basis of previous research, it was hypothesised that the occlusion of the mouth region by face masks would result in lower intensity ratings for intended emotions.

## Methods

### Participants

One-hundred and twenty adult participants (*M*_age_ = 33.48, *SD*_age_ = 10.81 years, 82 female) were recruited through Prolific (www.prolific.co). The study was created and hosted using the Gorilla Experiment Builder (www.gorilla.sc) [34] and participants completed the study using their own laptop or desktop computer. Data collection took place in February 2021. Participants were required to be between 18 and 60 years old, to have normal or corrected-to-normal vision, to have had no clinical diagnosis of autism spectrum disorder, and to have a Prolific study approval rate of 80% or higher. To ensure that participants had similar experiences of the COVID-19 pandemic and the associated use of face masks as a protective measure, participants were also required to be currently resident in the UK, and to not have travelled abroad in the previous 12 months. Six participants were replaced due to technical issues with image presentation: four reported that one or more of the images failed to load, and two showed problems with image calibration in the results output obtained from Gorilla.

The study was approved by the Departmental Ethics Committee for Psychological Sciences, Birkbeck, University of London, and was conducted in line with the ethical guidelines laid down in the 6th (2008) Declaration of Helsinki. Participants were presented with a digital consent form and provided informed consent by ticking a checkbox to confirm that they had read the form and ticking a second checkbox to confirm their agreement to participate. Participants were reimbursed for their time. The experimental task is available as Open Materials at gorilla.sc (https://app.gorilla.sc/openmaterials/241545). Data and a Matlab analysis script are available via the Open Science Framework (https://osf.io/mydgx/).

### Facial emotion rating task

The stimuli consisted of eight identities (four women, four men), each posing seven facial expressions: neutral, happiness, sadness, anger, fear, disgust, and surprise. Face stimuli were obtained from the Radboud Faces Database [35], a collection of facial images that are freely available for use in academic research (http://www.socsci.ru.nl:8180/RaFD2/RaFD). Each face was presented twice: once with a face mask superimposed over the nose and mouth, and once without a mask. Example stimuli can be viewed via the Open Science Framework (https://osf.io/mydgx/). There were 112 images in total (8 identities x 7 expressions x 2 mask conditions). Masks were superimposed using Adobe Photoshop. Images were cropped to include only the face and neck, and were presented at 4.8cm width (approximately 7cm height) on the participants’ screen.

Half of the participants (N = 60) saw the faces for 500ms, and the other half (N = 60) saw the faces for 3000ms. This manipulation was intended to reveal whether the detrimental effects of masks on expression interpretation varied as a function of presentation duration. Each participant viewed all 112 face images in a random order, with masked and unmasked faces intermixed. On each trial, the face image was preceded by a fixation cross (1000ms) and was replaced by a response screen. Participants rated the intensity of six emotions: happiness, sadness, anger, fear, disgust, and surprise, using six response sliders ranging from 0 (‘not at all’) to 100 (‘extremely’). All six sliders were presented on the screen at the same time. Participants were given unlimited time to respond. The order of these sliders was held constant throughout.

After completing the rating task, participants responded to two statements regarding their attitudes towards face masks: “Wearing a mask is an unpleasant experience”, and “People who wear masks in public places are silly”. Participants indicated their degree of agreement with each statement using a 5-point scale ranging from ‘strongly disagree’ (1) to ‘strongly agree’ (5). These questions were included to determine whether participants’ interpretation of masked and unmasked facial expressions was affected by attitudes to mask wearing. Finally, participants were asked whether they experienced any issues during the experiment.

## Results

### Emotion intensity ratings

**Fig 1:**
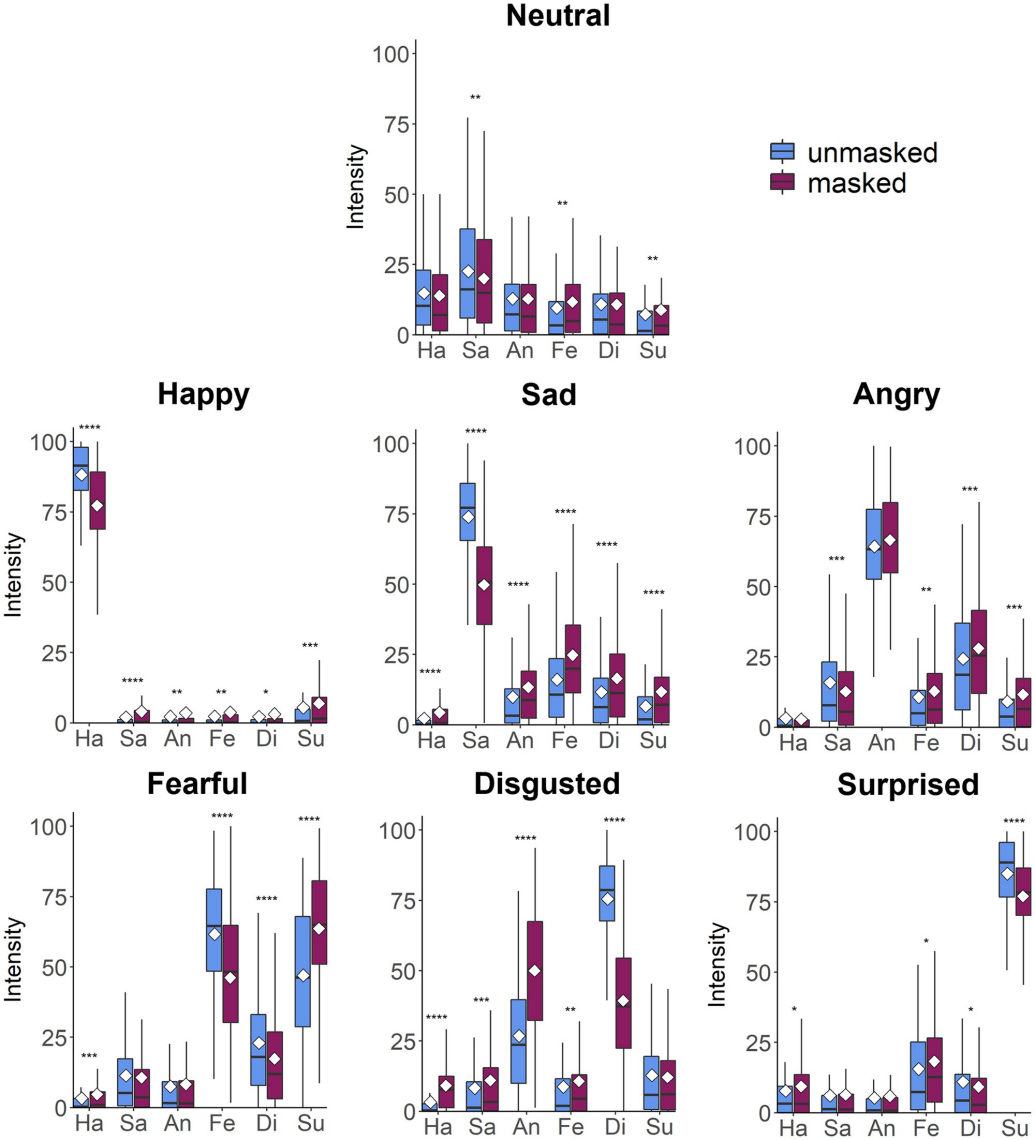
Intensity ratings for unmasked and masked faces. The mean of each distribution is depicted with a white square. The boxes show the median and the interquartile range. The whiskers show the furthest values that are within 1.5 x the interquartile range on either side of the box. Asterisks show significant contrasts between unmasked and masked ratings after FDR correction for six comparisons for each facial expression. Ha: happiness, Sa: sadness, An: anger, Fe: fear, Di: disgust, Su: surprise. * *p* ≤.05, ** *p* ≤.01, *** *p* ≤.001, **** *p* ≤.0001.

Emotion intensity ratings for unmasked and masked faces (Fig 1) were subjected to ANOVAs and planned paired *t*-tests (α = 0.05). All reported *p*-values are two-tailed. For each ANOVA where data violated the assumption of sphericity, we report Greenhouse-Geisser corrected degrees of freedom. For each paired t-test, Cohen’s *d* was calculated by dividing the mean difference by the standard deviation of the differences. Correction for multiple comparisons was performed using the false discovery rate (FDR). Although some of the rating distributions were approximately normal, others showed evidence of skewing due to the lower (0) and upper (100) bounds of the scale. ANOVA and *t*-tests have been shown to be robust to violations of normality [36, 37]. Nevertheless, we repeated all pairwise contrasts using Wilcoxon signed-rank tests, and we report these results in S1 Text. The results of these non-parametric analyses accorded closely with the parametric analyses described in the main text.

The intensity ratings obtained for each type of facial expression (neutral, happiness, sadness, anger, fear, disgust, and surprise) were initially subjected to separate ANOVAs with Presentation Duration (500ms, 3000ms) as a between-subjects factor and Rated Emotion (happiness, sadness, anger, fear, disgust, and surprise) and Mask Condition (unmasked, masked) as within-subject factors. We observed no main effects of Presentation Duration, nor any interactions with Presentation Duration, in any of these analyses (all *p*’s >.20). For the remaining analyses described below we therefore collapse across the two duration groups (N = 120).

The intensity ratings for each type of facial expression (neutral, happiness, sadness, anger, fear, disgust, and surprise) were subjected to separate ANOVAs with Rated Emotion (happiness, sadness, anger, fear, disgust, and surprise) and Mask Condition (unmasked, masked) as within-subject factors. The results of these analyses are summarised in Table 1. In each of these analyses, we observed significant main effects of Rated Emotion (all *p*’s < .001) and significant interactions between Rated Emotion and Mask Condition (all *p*’s < .001). There were also significant main effects of Mask Condition for happy, angry, and surprised expressions (all *p*’s < .05).

**Table 1:** Results of ANOVAs on the effects of Mask Condition and Rated Emotion on emotion intensity ratings.

Facial expression	Within-subject effects	Results			
		*df* (error)	F	*p*	η_p_^2^
Neutral	Mask Condition	1(119)	.000	.982	.000
	Rated Emotion	2.810 (334.363)	34.973	< .001	.227
	Mask Condition x Rated Emotion	3.982 (473.887)	5.897	< .001	.047
Happy	Mask Condition	1 (119)	4.028	.047	.033
	Rated Emotion	1.417 (168.629)	2654.935	< .001	.957
	Mask Condition x Rated Emotion	1.595 (189.779)	88.686	< .001	.427
Sad	Mask Condition	1 (119)	.009	.925	.000
	Rated Emotion	2.466 (293.450)	521.585	< .001	.814
	Mask Condition x Rated Emotion	2.641 (314.226)	147.177	< .001	.553
Angry	Mask Condition	1 (119)	14.378	< .001	.108
	Rated Emotion	2.847 (338.829)	470.458	< .001	.798
	Mask Condition x Rated Emotion	2.845 (338.606)	8.129	< .001	.064
Fearful	Mask Condition	1 (119)	1.937	.167	.016
	Rated Emotion	2.860 (340.363)	401.640	< .001	.771
	Mask Condition x Rated Emotion	2.422 (288.241)	80.511	< .001	.404
Disgusted	Mask Condition	1 (119)	1.807	.181	.015
	Rated Emotion	3.152 (375.029)	439.336	< .001	.787
	Mask Condition x Rated Emotion	2.026 (241.041)	257.500	< .001	.684
Surprised	Mask Condition	1 (119)	6.534	.012	.052
	Rated Emotion	2.519 (299.790)	1207.029	< .001	.910
	Mask Condition x Rated Emotion	3.163 (376.433)	24.146	< .001	.169

For unmasked faces, intensity ratings of the intended emotions were significantly higher than ratings of all non-intended emotions (all *p* < .001) for all facial expressions (happiness, sadness, anger, fear, disgust, and surprise) (Table 2). This was also the case for masked faces (*p*’s < .001), with the exception of fearful expressions (where ratings of surprise were significantly higher than ratings of fear, *p* < .001) and disgusted expressions (where ratings of anger were significantly higher than ratings of disgust, *p* < .001).

**Table 2:** Results of t-tests comparing intensity ratings of the intended emotion with each non-intended emotion (all *df*’s = 119, all *p*’s < .001).

Intended emotion	Non-intended emotions	Results			
		Unmasked		Masked	
		*t*	*D*	*t*	*d*
Happy	Sad	71.346	6.513	42.887	3.915
	Angry	67.686	6.179	43.836	4.002
	Fearful	71.258	6.505	42.923	3.918
	Disgusted	70.275	6.415	44.533	4.065
	Surprised	54.232	4.951	37.363	3.411
Sad	Happy	43.156	3.940	24.741	2.259
	Angry	32.421	2.960	17.818	1.627
	Fearful	26.170	2.389	11.370	1.038
	Disgusted	30.384	2.774	14.697	1.342
	Surprised	34.915	3.187	17.595	1.606
Angry	Happy	34.662	3.164	37.863	3.456
	Sad	22.185	2.025	25.568	2.334
	Fearful	26.224	2.394	25.967	2.370
	Disgusted	16.410	1.498	16.325	1.490
	Surprised	26.693	2.437	27.452	2.506
Fearful	Happy	30.669	2.800	19.332	1.765
	Sad	25.385	2.317	18.063	1.649
	Angry	27.046	2.469	17.812	1.626
	Disgusted	16.062	1.466	12.669	1.156
	Surprised	5.204	0.475	5.622 *	0.513
Disgusted	Happy	46.538	4.248	16.502	1.506
	Sad	36.472	3.329	14.756	1.347
	Angry	20.346	1.857	3.813 *	0.348
	Fearful	36.287	3.313	16.378	1.495
	Surprised	31.565	2.881	16.032	1.464
Surprised	Happy	50.112	4.575	40.608	3.707
	Sad	50.297	4.592	44.735	4.084
	Angry	51.438	4.696	43.517	3.973
	Fearful	33.936	3.098	28.565	2.608
	Disgusted	39.990	3.651	38.291	3.495

Ratings of intended emotions were higher than ratings of non-intended emotions, except for the two cases marked with asterisks, for which ratings of the non-intended emotions were significantly higher than ratings of the intended emotion.

**Fig 2:**
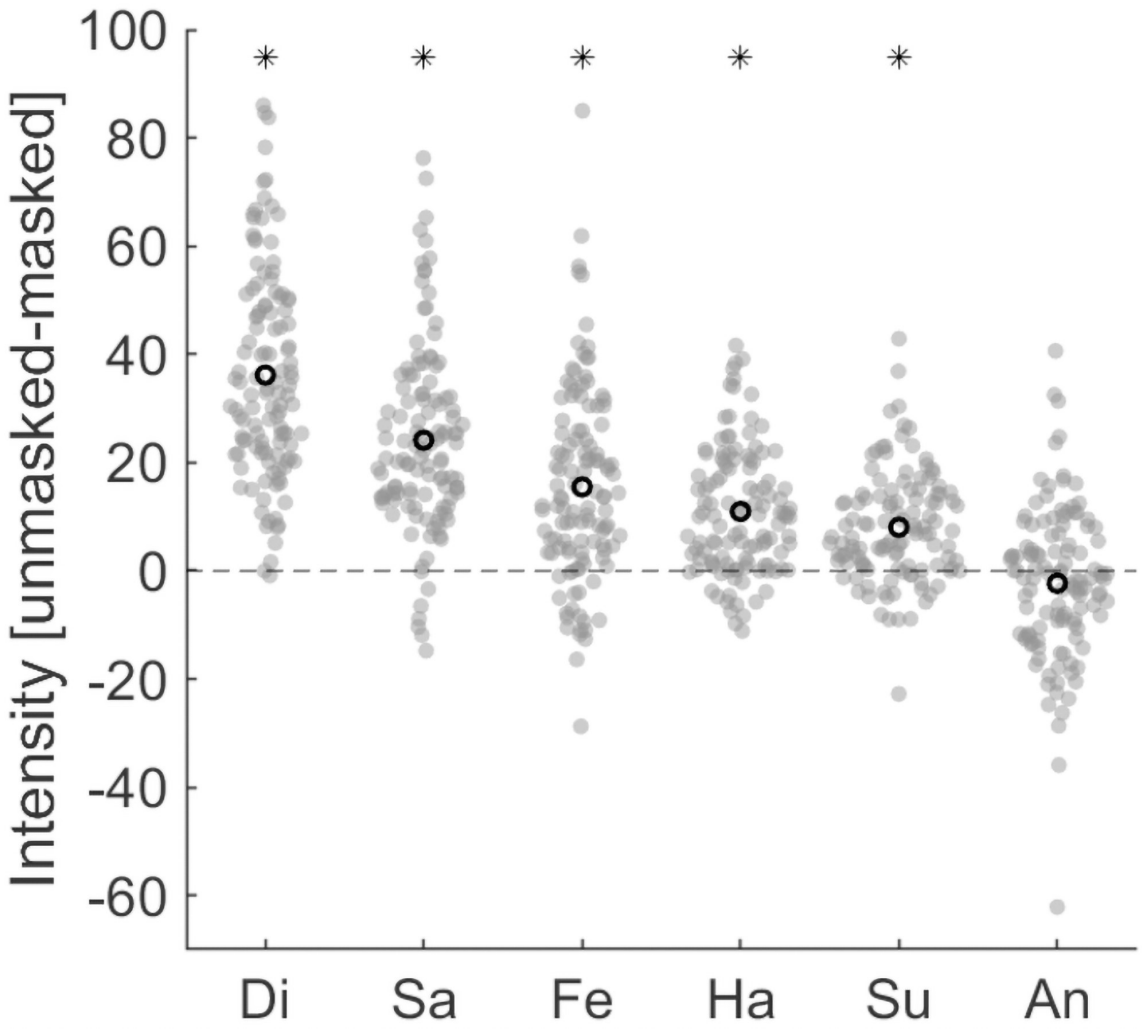
Differences in intensity ratings of intended emotions between unmasked and masked faces. Black hollow circles show the means. Positive values indicate higher ratings for unmasked faces. Emotions are sorted from highest to lowest based on the mean difference. Asterisks show significant contrasts between unmasked and masked ratings after FDR correction for six comparisons. Ha: happiness, Sa: sadness, An: anger, Fe: fear, Di: disgust, Su: surprise.

Mean intensity ratings of the intended emotions of unmasked and masked faces are shown in Table 3. The perceived intensity of the intended emotion was significantly lower when faces were masked compared to when they were unmasked for expressions of happiness [*t*(119) = 10.332, *p* < .001, *d* = 0.943], sadness [*t*(119) = 15.376, *p* < .001, *d* = 1.404], fear [*t*(119) = 9.480, *p* < .001, *d* = 0.865], disgust [*t*(119) = 20.551, *p* < .001, *d* = 1.876], and surprise [*t*(119) = 8.707, *p* < .001, *d* = 0.795], but not for anger [*t*(119) = 1.811, *p* = .073, *d* = 0.165] (subject to FDR correction for six comparisons). The difference in the perceived intensity of the intended emotion between unmasked and masked faces was highest for expressions of disgust (*M* = 36.16, 95% CI [32.68, 39.65], *SD* = 19.28, *Mdn* = 32.88), followed by sadness (*M* = 24.13, 95% CI [21.02, 27.23], *SD* = 17.19, *Mdn* = 20.88), fear (*M* = 15.50, 95% CI [12.26, 18.73], *SD* = 17.91, *Mdn* = 13.44), happiness (*M* = 10.96, 95% CI [8.86, 13.05], *SD* = 11.61, *Mdn* = 10.06), and surprise (*M* = 8.04, 95% CI [6.21, 9.87], *SD* = 10.12, *Mdn* = 6.81) (Fig 2).

**Table 3:** Descriptive statistics for intensity ratings of the intended emotions of unmasked and masked faces.

		M	95% CI	SD	Mdn
Unmasked	Happy	88.26	[86.11, 90.41]	11.89	91.44
	Sad	73.91	[70.80, 77.02]	17.20	77.19
	Angry	64.28	[60.86, 67.69]	18.91	63.25
	Fearful	61.66	[57.92, 65.40]	20.69	64.56
	Disgusted	75.54	[72.57, 78.51]	16.44	78.69
	Surprised	85.04	[82.62, 87.45]	13.36	88.94
Masked	Happy	77.31	[74.35, 80.27]	16.37	78.13
	Sad	49.78	[46.21, 53.36]	19.78	50.81
	Angry	66.59	[63.30, 69.87]	18.19	66.13
	Fearful	46.17	[41.83, 50.51]	24.01	48.31
	Disgusted	39.38	[35.67, 43.09]	20.52	37.31
	Surprised	76.99	[74.47, 79.52]	13.98	78.31

The perceived intensity of non-intended emotions tended to be higher when faces were masked compared to when they were unmasked. To compare ratings of non-intended emotions across masked and unmasked faces, for each emotional expression we averaged across ratings of all five non-intended emotions. Ratings of non-intended emotions were significantly higher for masked faces compared with unmasked faces for expressions of happiness [*t*(119) = 4.009, *p* < .001, *d* = 0.366], sadness [*t*(119) = 10.222, *p* < .001, *d* = 0.933], anger [*t*(119) = 2.317, *p* = .022, *d* = 0.212], fear [*t*(119) = 5.729, *p* < .001, *d* = 0.523], and disgust [*t*(119) = 14.385, *p* < .001, *d* = 1.313], but not for surprise [*t*(119) = 1.847, *p* = .067, *d* = 0.169] (subjected to FDR correction for six comparisons).

For neutral expressions, intensity ratings for sadness were higher than ratings of all other emotions for both unmasked and masked faces (all *p*’s < .001; full results are presented in S1 Table). Unmasked neutral faces received higher intensity ratings for sadness than masked neutral faces [*t*(119) = 2.920, *p* = .004, *d* = 0.267].

### Confusion patterns

To determine whether the confusion patterns of different emotions are altered by mask wearing, we compared the reliability of emotion intensity ratings across mask conditions with the reliability of ratings within mask conditions. Specifically, we constructed confusion matrices from the emotion intensity ratings, and computed the reliability of the confusion matrices across unmasked and masked versions of the same faces, and compared it with the reliability of confusion matrices (a) within unmasked faces, and (b) within masked faces. This approach has been used previously to compare the reliability of emotion confusion patterns across different sensory modalities with reliability within modalities [38]. If confusion patterns are altered by mask wearing, we would expect the reliability of confusion patterns across mask conditions to be lower than the reliability within mask conditions.

**Fig 3:**
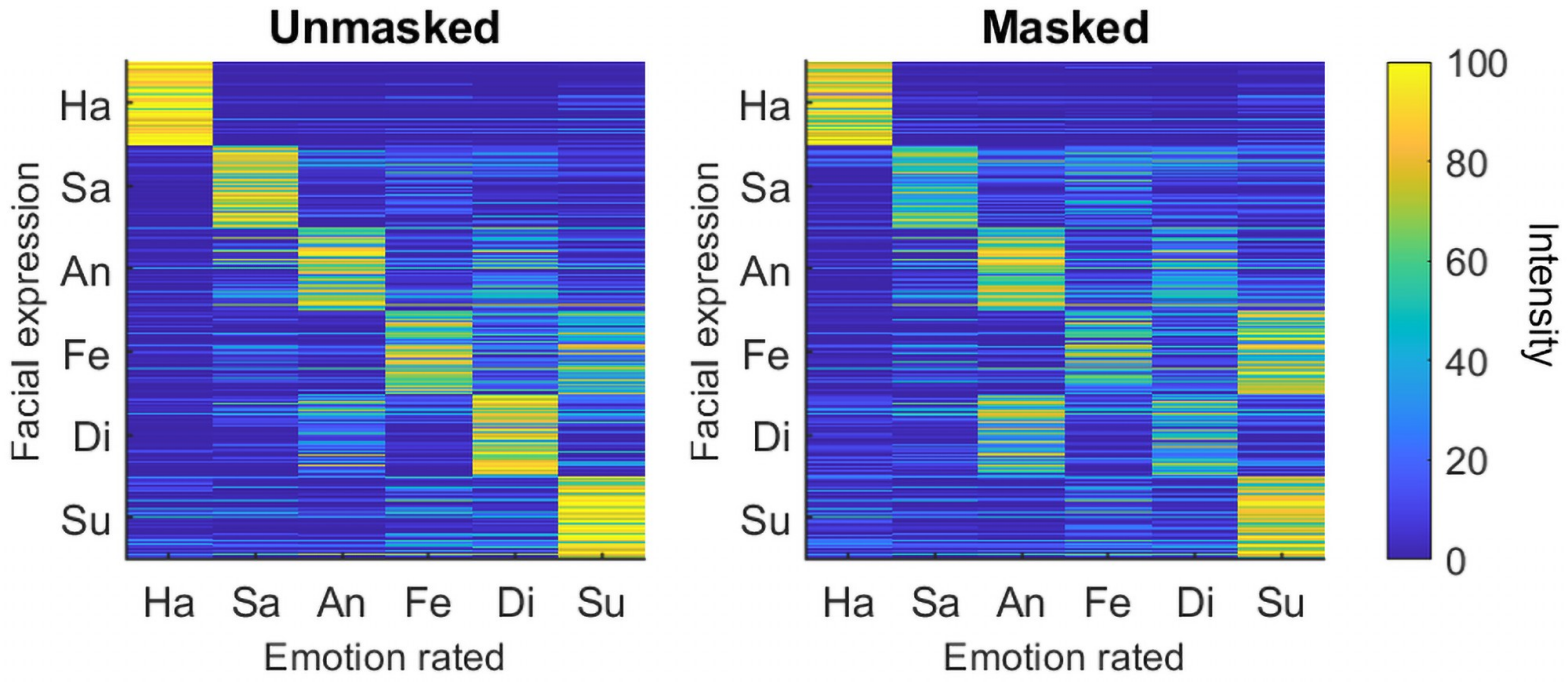
Confusion matrices showing participants’ emotion intensity ratings for each emotional expression (excluding neutral). Individual participant ratings for each facial expression are presented in individual rows, and the rated emotion is indicated by the columns.

To create confusion matrices, for each participant, and for each of the two mask conditions (unmasked, masked) separately, the data was split into two subsets: data for identities 1, 3, 5, and 7 were in subset A, and data for identities 2, 4, 6, and 8 were in subset B. Each subset contained two female and two male identities. For each subset, a 6 x 6 confusion matrix was computed by averaging the intensity ratings for each facial expression (excluding neutral) across the four identities, and arranging the ratings so that rows represented the facial expressions, and columns represented the emotions rated (in the format presented in Fig 3). This resulted in four confusion matrices for each participant: (1) unmasked subset A, (2) unmasked subset B, (3) masked subset A, and (4) masked subset B. For the analysis, the matrices were transformed to vector format, and raw correlations were subjected to a Fisher z-transformation to approximate a normal distribution. Split-half reliability *within* mask conditions was computed as the Spearman correlation between the ratings in subsets A and B for unmasked faces, and for masked faces. Split-half reliability *across* mask conditions was computed as the correlation between subset A for unmasked faces and subset B for masked faces.

Mean split-half reliability was highest in the unmasked condition (mean *r*_s_ = .82), followed by the masked condition (mean *r*_s_ = .77), and was lowest across mask conditions (mean *r*_s_ = .74). Paired t-tests showed that the emotion confusion patterns were less reliable across mask conditions than within the unmasked condition [*t*(119) = 7.871, *p* < .001, *d* = 0.719] and within the masked condition [*t*(119) = 3.637, *p* < .001, *d* = 0.332]. Patterns were also less reliable in the masked condition compared with the unmasked condition [*t*(119) = 4.822, *p* < .001, *d* = 0.440].

### Attitudes towards masks

The mean agreement rating for the statement: “Wearing a mask is an unpleasant experience” was 3.24 (*SD* = 1.08). The mean agreement rating for the statement: “People who wear masks in public places are silly” was 1.2 (*SD* = 0.60). Both ratings were based on a 5-point scale with higher ratings indicating stronger agreement. Correlational analyses were conducted with the difference scores illustrated in Fig 2 (i.e., the emotion intensity ratings of intended emotions in the unmasked condition less the emotion intensity ratings of intended emotions in the masked condition).

In the combined sample (N = 120), there were no significant correlations between the difference scores and agreement with either statement for any of the emotions (all *r*_s_ < .18, *p* >.05). This was also the case when the data from the 3000ms duration condition (N = 60) was analysed separately (all *r*_s_ < .23, *p* >.07). In the 500ms condition (N = 60), there was a weak correlation between difference scores for happy faces and responses to the statement “Wearing a mask is an unpleasant experience” (*r*_s_ = .26, *p* = .046); however, this relationship did not survive FDR correction for six comparisons. No other correlations were significant in the 500ms condition (*r*_s_ < .25, *p* >.06).

## Discussion

The present study investigated how the partial occlusion of a face with a surgical-type mask affects the perceived emotional profile of facial expressions of happiness, sadness, anger, fear, disgust, and surprise. We found that masks reduced the perceived intensity of intended emotions, for all expressions except for anger. Additionally, masks increased the perceived intensity of non-intended emotions for all expressions except surprise. In the mask condition, the intended emotions were perceived as more intense than all non-intended emotions for happy, sad, angry, and surprised faces. However, masked disgusted faces received higher ratings for angry, and masked fearful faces received higher ratings for surprise. Rating patterns were more reliable within mask conditions (unmasked, masked) than across mask conditions, suggesting that masks alter the overall pattern of confusion between different emotions.

Our finding of reduced perceived intensity of intended emotions for masked faces is in line with previous studies showing negative effects of mask-wearing on emotion categorisation accuracy [23, 24, 27, 28], and underscores the importance of the mouth region for emotion recognition [14, 15, 20, 22]. However, angry expressions were an exception to this pattern: We found that mask-wearing had no effect on the perceived intensity of the target emotion. The robustness of anger perception to occlusions of the lower face is supported by findings showing no difference in the recognition of anger between faces of individuals wearing a niqab, where only the eyes are visible, and uncovered faces [39, 40]. We also note that the recognition of anger is relatively unaffected by the presence of sunglasses [24]. The social importance of angry expressions as a means to communicate and detect threat may explain why anger is detectable across the entire face.

The presence of a mask not only led to a decrease in perceived intensity of the intended emotions, but it also resulted in an overall increase in the perceived intensity of non-intended emotions (with the exception of surprised expressions). This increase was most striking for fearful and disgusted expressions, for which the perceived intensity of the non-intended emotions of surprise and anger, respectively, was greater than the perceived intensity of the intended emotions. Disgust and fear, along with sadness, also showed the greatest decreases in perceived target-emotion intensity in the masked condition. A recent study employing an emotion-categorisation task with masked faces showed that disgusted faces were mislabelled as angry, and fearful faces were mislabelled as surprised [24], suggesting that these confusions are not specific to our stimuli or task.

Confusions between fear and surprise, and between disgust and anger, have been previously associated with a tendency to focus on the eye region over the mouth region [41]. Nevertheless, the mouth region clearly provides important diagnostic information that enables observers to distinguish fear from surprise, and disgust from anger [13, 41–43]. Where confusions arise (e.g., between fear and surprise, and between disgust and anger), there are likely to be implications for social interaction; for example, the observer may respond inappropriately, leading to misunderstanding and conflict. When confronted with the similarity between anger and disgust in the mask condition, participants were more likely to perceive anger in both types of stimulus. Similarly, participants were more likely to perceive surprise when confronted with the similarity between fear and surprise. We speculate these biases reflect the greater likelihood of encountering anger and surprise in our day-to-day interactions, compared to disgust and fear.

Masked expressions of happiness, sadness, anger, and surprise received higher intensity ratings for intended emotions, than for non-intended emotions. This suggests that people are quite good at recognising these emotions in masked faces, even after just half a second of exposure to the face. It is possible that these expressions are more easily identifiable from the top half of the face alone, compared to fear and disgust. Another possibility is that these emotions are more commonly encountered during day-to-day interactions, and the ability to recognise them in masked faces could be a sign of emerging expertise in deciphering the facial expressions of mask wearers. A recent pre-print study found a relationship between the degree of exposure to masked faces and the use of cues in the eye region when judging the perceived similarity of emotional faces [44], suggesting that masks may be changing the way in which facial expressions are processed.

As expected, mean emotion intensity ratings for neutral faces were low for all emotions across both masked and unmasked faces (<30%). However, intensity ratings for sadness were higher than ratings of all other emotions for both unmasked and masked faces, with the presence of a mask reducing the perceived intensity of sadness. Confusion of neutral masked faces for sad was also found in a study employing an emotion categorisation paradigm [28]. These results are in line with previous studies showing that neutral facial expressions tend to be perceived as negative, rather than truly ‘neutral’ [45–47].

Previous studies have reported a drop in emotion categorisation accuracy when the lower halves of target faces were occluded by face masks [23, 24, 27]. The duration of stimulus presentation in these studies was either set to one second [24, 27], or was unlimited [23]. Because, these authors used different stimuli and different methods, it is difficult to assess the potential impact of differences in presentation duration. In the present study, we were able to manipulate presentation duration using the same stimuli and the same task. However, we found no effect of presentation duration (500ms or 3000ms) on intensity ratings for any emotion, in either mask condition. In light of these findings, it seems unlikely that differences in presentation duration affected the previous results described using categorisation paradigms [23, 24, 27].

Previous research suggests that the interpretation of facial emotion can be affected by mood [48, 49] and expectations [50, 51]. We were therefore interested in the possibility that attitudes towards mask wearing and mask wearers might affect the emotions attributed in our rating task. However, we found no relationship between participants’ experiences of wearing a face mask, or their attitude towards mask wearers, and their emotion intensity ratings. We note however, the vast majority of participants showed a positive attitude towards people who wear masks; for example, 87% of respondents strongly disagreed with the statement “People who wear masks in public places are silly”. This lack of variability may have hindered our ability to detect a potential relationship between task performance and agreement with this statement. Future research might also examine whether positively phrased statements (e.g., “People who wear masks in public places are responsible and considerate”) reveal a relationship between expression interpretation and attitudes to mask wearing.

In the present study, face masks were superimposed over images of emotional faces, as in previous studies [23, 24, 27–29]. While this treatment maximises the chances that any differences between masked and unmasked faces are due to the presence of the mask, it is important to consider the possibility that the appearance of facial expressions may differ when wearing a mask, compared to when not wearing a mask. Responses to a UK survey on the impact of face coverings on communication revealed conscious attempts to convey emotion using the eyes, such as “smiling with the eyes”, and attempts to exaggerate facial expressions when wearing a mask [26]. It remains unclear whether these strategies are effective–people have relatively poor insight into the appearance of their facial gestures and expressions [52]. It is possible, however, that findings obtained with artificially-masked faces underestimate the ability of people to interpret the expressions of mask wearers outside the lab.

Online testing has been a great innovation that can help researchers achieve larger sample sizes. Evidence suggests that this approach can produce high-quality data comparable with that obtained from in-person testing [53–55]. To give recent examples from our own research, we have found that online testing has produced clear, replicable results in visual search [56, 57] and attention cueing [58, 59] experiments, and studies of visual illusions [60, 61]. However, we acknowledge that this approach is associated with some well-known limitations [25]. For example, it is not easy to control the testing environment, the participants’ viewing distance, or their monitor settings. Finally, we note that our results apply to Caucasian faces and predominantly female observers from the UK population. Consequently, it remains unclear how well these findings generalise well to faces of other ethnicities and different populations [25].

To summarize, the present work examined how the presence of face masks affect the interpretation of prototypical facial expressions. The presence of a mask tended to lower the perceived intensity of intended emotions (with the exception of angry expressions), and increase the perceived intensity of unintended emotions (with the exception of surprised expressions). The presence of a face mask led participants to confuse disgust for anger, and fear for surprise. With the use of face masks likely to remain an important measure in containing the spread of COVID-19 in the foreseeable future, it will be interesting to observe how people adapt to this new barrier to non-verbal communication.

## Supporting information

S1 Table Results of t-tests for neutral faces comparing intensity ratings of sadness with every other emotion (all *df*’s = 119, all *p*’s < .001). (DOCX)

S1 Text Non-parametric analysis. (DOC)
