# Supplementary material for: How does the presence of a surgical face mask impair the perceived intensity of facial emotions?
Source: PLoS One. 2022 Jan 13;17(1):e0262344. doi: 10.1371/journal.pone.0262344 (PMC8758043; doi:10.1371/journal.pone.0262344)
Supplement: S1 Table — (DOCX) [file pone.0262344.s001.docx]

**How does the presence of a surgical face mask impair the perceived intensity of facial emotions?**

**Maria Tsantani, Vita Podgajecka, Katie L. H. Gray, Richard Cook**

**Supporting information**

**S1 Table.** **Results of t-tests for neutral faces comparing intensity ratings of sadness with every other emotion (all *df*’s = 119, all *p*’s <.001).**

|  | Results | | | | |
| --- | --- | --- | --- | --- | --- |
|  | Unmasked | | | Masked | |
|  | *t* | *d* | *t* | | *d* |
| Happy | 4.710 | .430 | 4.639 | | .423 |
| Angry | 6.050 | .552 | 6.012 | | .549 |
| Fearful | 8.699 | .794 | 7.131 | | .651 |
| Disgusted | 7.557 | .690 | 7.460 | | .681 |
| Surprised | 9.402 | .858 | 8.501 | | .776 |
