# Supplementary material for: How does the presence of a surgical face mask impair the perceived intensity of facial emotions?
Source: PLoS One. 2022 Jan 13;17(1):e0262344. doi: 10.1371/journal.pone.0262344 (PMC8758043; doi:10.1371/journal.pone.0262344)
Supplement: S1 Text — (DOC) [file pone.0262344.s002.doc]

**How does the presence of a surgical face mask impair the perceived intensity of facial emotions?**

**Maria Tsantani, Vita Podgajecka, Katie L. H. Gray, Richard Cook**

**Supporting information**

**Non-parametric analysis**

In addition to the paired t-tests described in the manuscript, we repeated all analyses of pairwise contrasts using Wilcoxon signed-rank tests, which do not assume that data is normally distributed. Effect sizes for the resulting *Z* statistics (*r*) were calculated by dividing *Z* by the square root of the number of observations.

For unmasked faces, intensity ratings of the intended emotions were significantly higher than ratings of all non-intended emotions (all *p* <.001) for all facial emotions (happy, sad, angry, fearful, disgusted, and surprised) (Table A). This was also the case for masked faces (*p*’s < .001), with the exception of fearful expressions (where ratings of surprise were significantly higher than ratings of fear, *p* < .001) and disgusted expressions (where ratings of anger were significantly higher than ratings of disgust, *p* < .001).

Intensity ratings of the intended emotions were significantly lower when faces were masked compared to when they were unmasked for expressions of happiness [*Z* = 8.028, *p* < .001, *r* = .518], sadness [*Z* = 9.233, *p* < .001, *r* = .596], fear [*Z* = 7.658, *p* < .001, *r* = .494], disgust [*Z* = 9.464, *p* < .001, *r* = .611], and surprise [*Z* = 7.311, *p* < .001, *r* = .472], but not for anger [Z = 1.892, *p* = .059, *r* = .122] (subject to FDR correction for six comparisons).

Ratings of non-intended emotions (averaged across all five non-intended emotions) were significantly higher for masked faces compared with unmasked faces for expressions of happiness [*Z* = 5.446, *p* < .001, *r* = .352], sadness [*Z* = 8.397, *p* < .001, *r* = .542], anger [*Z* = 2.462, *p* = .014, *r* = .159], fear [*Z* = 5.284, *p* < .001, *r* = .341], and disgust [*Z* = 8.807, *p* < .001, *r* = .569], but not for surprise [*Z* = 1.605, *p* = .109, *r* = .104] (subjected to FDR correction for six comparisons).

For neutral expressions, intensity ratings for sadness were higher than ratings of all other emotions for both unmasked and masked faces (all *p*’s < .001). Full results are shown in Table B. Unmasked neutral faces received higher intensity ratings for sadness than masked neutral faces [*Z* = 2.637, *p* = .008, *r* = .170].

**Table A.** **Results of Wilcoxon signed-rank tests comparing intensity ratings of the intended emotion with each non-intended emotion (all *p*’s < .001).**

| Intended emotion | Non-intended emotions | Results | | | | |  |
| --- | --- | --- | --- | --- | --- | --- | --- |
|  |  | Unmasked | | Masked | | |  |
|  |  | *Z* | *r* | | *Z* | *r* | |
| Happy | Sad | 9.508 | .614 | | 9.507 | .614 | |
|  | Angry | 9.508 | .614 | | 9.507 | .614 | |
|  | Fearful | 9.508 | .614 | | 9.507 | .614 | |
|  | Disgusted | 9.508 | .614 | | 9.507 | .614 | |
|  | Surprised | 9.508 | .614 | | 9.507 | .614 | |
| Sad | Happy | 9.507 | .614 | | 9.464 | .611 | |
|  | Angry | 9.501 | .613 | | 9.207 | .594 | |
|  | Fearful | 9.496 | .613 | | 8.199 | .529 | |
|  | Disgusted | 9.507 | .614 | | 8.864 | .572 | |
|  | Surprised | 9.507 | .614 | | 9.215 | .595 | |
| Angry | Happy | 9.507 | .614 | | 9.507 | .614 | |
|  | Sad | 9.507 | .614 | | 9.493 | .613 | |
|  | Fearful | 9.507 | .614 | | 9.507 | .614 | |
|  | Disgusted | 9.463 | .611 | | 9.432 | .609 | |
|  | Surprised | 9.499 | .613 | | 9.507 | .614 | |
| Fearful | Happy | 9.507 | .614 | | 9.457 | .610 | |
|  | Sad | 9.507 | .614 | | 9.438 | .609 | |
|  | Angry | 9.499 | .613 | | 9.412 | .608 | |
|  | Disgusted | 9.103 | .588 | | 8.799 | .568 | |
|  | Surprised | 4.916 | .317 | | 5.350* | .345 | |
| Disgusted | Happy | 9.507 | .614 | | 9.420 | .608 | |
|  | Sad | 9.507 | .614 | | 9.112 | .588 | |
|  | Angry | 9.433 | .609 | | 3.712* | .240 | |
|  | Fearful | 9.507 | .614 | | 9.326 | .602 | |
|  | Surprised | 9.507 | .614 | | 9.300 | .600 | |
| Surprised | Happy | 9.507 | .614 | | 9.507 | .614 | |
|  | Sad | 9.507 | .614 | | 9.507 | .614 | |
|  | Angry | 9.507 | .614 | | 9.507 | .614 | |
|  | Fearful | 9.507 | .614 | | 9.507 | .614 | |
|  | Disgusted | 9.507 | .614 | | 9.507 | .614 | |

Ratings of intended emotions were higher than ratings of non-intended emotions, except for the two cases marked with asterisks, for which ratings of the non-intended emotions were significantly higher than ratings of the intended emotion.

**Table B.** **Results of Wilcoxon signed-rank for neutral faces comparing intensity ratings of sadness with every other emotion (*p*’s <.001).**

|  | Results | | | | |
| --- | --- | --- | --- | --- | --- |
|  | Unmasked | | | Masked | |
|  | *Z* | *r* | *Z* | | *r* |
| Happy | 4.644 | .300 | 4.919 | | .318 |
| Angry | 5.942 | .384 | 5.790 | | .374 |
| Fearful | 8.030 | .518 | 6.567 | | .424 |
| Disgusted | 6.906 | .446 | 6.990 | | .451 |
| Surprised | 8.396 | .542 | 7.407 | | .478 |
